# Flotillins Interact with PSGL-1 in Neutrophils and, upon Stimulation, Rapidly Organize into Membrane Domains Subsequently Accumulating in the Uropod

**DOI:** 10.1371/journal.pone.0005403

**Published:** 2009-04-30

**Authors:** Jérémie Rossy, Dominique Schlicht, Britta Engelhardt, Verena Niggli

**Affiliations:** 1 Department of Pathology, University of Bern, Bern, Switzerland; 2 Theodor Kocher Institute, University of Bern, Bern, Switzerland; Emory University, United States of America

## Abstract

**Background:**

Neutrophils polarize and migrate in response to chemokines. Different types of membrane microdomains (rafts) have been postulated to be present in rear and front of polarized leukocytes and disruption of rafts by cholesterol sequestration prevents leukocyte polarization. Reggie/flotillin-1 and -2 are two highly homologous proteins that are ubiquitously enriched in detergent resistant membranes and are thought to shape membrane microdomains by forming homo- and hetero-oligomers. It was the goal of this study to investigate dynamic membrane microdomain reorganization during neutrophil activation.

**Methodology/Principal Findings:**

We show now, using immunofluorescence staining and co-immunoprecipitation, that endogenous flotillin-1 and -2 colocalize and associate in resting spherical and polarized primary neutrophils. Flotillins redistribute very early after chemoattractant stimulation, and form distinct caps in more than 90% of the neutrophils. At later time points flotillins accumulate in the uropod of polarized cells. Chemotactic peptide-induced redistribution and capping of flotillins requires integrity and dynamics of the actin cytoskeleton, but does not involve Rho-kinase dependent signaling related to formation of the uropod. Both flotillin isoforms are involved in the formation of this membrane domain, as uropod location of exogenously expressed flotillins is dramatically enhanced by co-overexpression of tagged flotillin-1 and -2 in differentiated HL-60 cells as compared to cells expressing only one tagged isoform.

Flotillin-1 and -2 associate with P-selectin glycoprotein ligand 1 (PSGL-1) in resting and in stimulated neutrophils as shown by colocalization and co-immunoprecipitation. Neutrophils isolated from PSGL-1-deficient mice exhibit flotillin caps to the same extent as cells isolated from wild type animals, implying that PSGL-1 is not required for the formation of the flotillin caps. Finally we show that stimulus-dependent redistribution of other uropod-located proteins, CD43 and ezrin/radixin/moesin, occurs much slower than that of flotillins and PSGL-1.

**Conclusions/Significance:**

These results suggest that flotillin-rich actin-dependent membrane microdomains are importantly involved in neutrophil uropod formation and/or stabilization and organize uropod localization of PSGL-1.

## Introduction

An adequate innate immune response requires neutrophils to rapidly bind to and transmigrate through endothelial cells, and chemotax through the extracellular matrix toward the source of inflammation. Concomitant with binding to the vascular endothelium, neutrophils are activated by a combination of adhesion-triggered signaling and chemokine-dependent stimulation [1], [2]. Activated neutrophils become polarized with a contracted tail (uropod) in the rear and F-actin-rich protrusions at the front and start crawling. Actin and proteins regulating actin polymerization are key players in the establishment of the morphological and functional cell polarity. Actin polymerization and membrane ruffling are the first events leading to establishment of chemoattractant-stimulated neutrophil polarization [3]. Phosphatidylinositol 3-kinase, together with Rac and Cdc42 organize F-actin and membrane protrusion in the leading edge, whereas the Rho/ROCK pathway governs acto-myosin contraction and rear detachment [4], [5].

Membrane microdomains also appear to contribute to shaping and sustaining cell polarity. Indeed, treatment of neutrophils or neutrophil-like HL-60 cells with methyl-β-cyclodextrin, a cyclic oligosaccharide used to deplete membrane cholesterol and to disrupt cholesterol-rich membrane microdomains, prevents stimulus-induced actin polymerization, polarization and chemokinesis [6], [7]. Furthermore, it has been shown that proteins retrieved in detergent resistant membranes (DRMs) segregate in two opposite “sets” of membrane microdomains, situated at the leading and trailing edges of polarized leukocytes [8], [9], [10].

Membrane microdomains located in the uropod of neutrophils contain transmembrane adhesion proteins such as P-selectin glycoprotein ligand-1 (PSGL-1), L-selectin, CD43 or CD44 [8], [11]. Importantly, disruption of the actin cytoskeleton prevents redistribution of those membrane microdomain proteins [12], [13], demonstrating that their localization in the uropod of leukocytes depends on an intact actin network. According to two recent publications, cholesterol-rich microdomains in the plasma membrane are linked to the actin cytoskeleton and depend on it for their existence and activation-induced coalescence [14], [15]. Thus, proteins residing in membrane microdomains and able to link to the actin cytoskeleton might be important for segregating transmembrane adhesion proteins into the uropod of leukocytes. Indeed, ezrin/radixin/moesin (ERM) proteins involved in actin-membrane linkage, have also been detected in leukocyte uropod rafts [16].

Reggie/flotillin-1 and -2 are two highly homologous proteins whose enrichment in DRMs has been ubiquitously observed. Yet, their function remains unclear [17], [18]. Nevertheless, several publications suggest that flotillins play a role in cell adhesion and migration. Reduction of flotillin-1 expression in lymphocytes reduces chemotaxis and adhesion [19], misexpression of flotillin in Drosophila embryos impairs the localization of intercellular adhesion molecules [20] and overexpression or silencing of flotillin-2 respectively increases or decreases cell spreading [21]. Flotillins can be responsible for creating or scaffolding membrane microdomains, thanks to their ability to form large multimeric complexes [17]. They are able to connect to the actin cytoskeleton, indirectly via the cytoskeletal protein vinexin [22] or by direct interaction with actin [23]. Interestingly, flotillins are upregulated during neutrophilic differentiation of HL-60 cells, indicating important roles of these proteins in neutrophil-specific functions [24].

It was the aim of this study to visualize membrane microdomain/raft dynamics during neutrophil activation, using as a raft marker flotillin-1 and -2. We show here that endogenous flotillin-1 and -2 interact and upon stimulation with chemotactic peptide rapidly form membrane caps and at later time points accumulate in the uropods of polarized cells in more than 90% of primary neutrophils. Both flotillin-1 and flotillin-2 are involved in formation of this membrane domain, as uropod location of exogenously expressed flotillins is dramatically enhanced by co-overexpression of tagged flotillin-1 and -2 in dHL-60 cells as compared to cells expressing only one tagged isoform. Capping of endogenous flotillins in human neutrophils stimulated with chemotactic peptides is sensitive to disruption or stabilization of actin cytoskeleton whereas inhibition of acto-myosin signaling implied in uropod formation has no impact. Additionally, we present evidence showing that, during polarization of human neutrophils, flotillin capping precedes capping of other uropod components such as CD43 and phosphorylated ERM proteins (P-ERM). Finally we show that flotillin capping and redistribution to the uropod matches temporally and spatially that of PSGL-1, and that flotillins interact (directly or indirectly) with PSGL-1 in primary human neutrophils. Using neutrophils isolated from PSGL-1 deficient mice, we show that PSGL-1 is not required for the capping of flotillins. This study suggests novel roles of flotillin isoforms in neutrophil polarization and adhesion protein relocation.

## Methods

### Materials

Materials and suppliers: N-formyl-L-norleucyl-L-leucyl-L-phenylalanyl-L-norleucyl-L-tyrosyl-L-lysine (fNLPNTL), Bachem, Bubendorf, Switzerland. Y-27632, Jasplakinolide: Calbiochem, La Jolla, CA, USA. Latrunculin A, ML-7: Alexis Biochemicals, Lausen, Switzerland. Blebbistatin: Tocris Bioscience, Bristol, UK. Recombinant murine G-CSF: Preprotech, London, England. OptiPrep: Axis-Shield, Oslo, Norway. Percoll and protein A or G-coupled beads: GE Healthcare Bio-Sciences AB, Uppsala, Sweden. Bovine serum albumin (BSA): Serva, Heidelberg, Germany. Lysolecithin (L-α-lysophosphatidylcholine), DMSO, Histopaque 1083 and Methocel (MC, low viscosity): Sigma, St. Louis, MO, USA. Protease inhibitor cocktail pH 8: Roche Diagnostics AG, Rotkreuz, Switzerland. Hank's buffered salt solution (HBSS) was from Applichem, Darmstadt, Germany. Gey's solution contained 138 mM NaCl, 6 mM KCl, 1 mM MgSO_4_, 1.1 mM CaCl_2_, 100 µM EGTA, 1 mM Na_2_HPO_4_, 5 mM NaHCO_3_, 5.5 mM glucose and 20 mM HEPES (pH 7.4).

### Antibodies

The monoclonal rat anti-mouse PSGL-1 (4RB12) was prepared as described [25]. Polyclonal anti-phospho Ezrin (Thr567)/Radixin (Thr564)/Moesin (Thr558) antibody (Cat. No. 3141S) was from Cell Signaling, Beverly, MA, USA. Polyclonal anti-flotillin-2 (Cat. No. sc-25507) was from Santa Cruz Biotechnology, Santa Cruz, CA, USA. A monoclonal anti-actin antibody (Cat. No. 010056) was from Bio-Science Products AG Emmenbrücke, Switzerland. Monoclonal murine antibodies directed against CD43 (Cat. No. 551457), flotillin-1 (Cat. No. F65020), flotillin-2 (Cat. No. F35820) and PSGL-1 (Cat. No. 556053) were obtained from Transduction Laboratories/BD Pharmingen, Heidelberg, Germany. The Alexa 488-conjugated goat-anti-rabbit (Cat. No. A11008), Alexa-568-conjugated goat anti-rat (Cat. No. A11077) and goat anti-mouse IgG antibodies (Cat. No. A10001) were from Molecular Probes, Eugene, OR, USA. The goat-anti-rabbit and goat-anti-mouse IgG horseradish peroxidase conjugated antibodies (Cat. No. 170-6515 and 170-65-16) were obtained from BioRad, Hercules, CA, USA. Murine control IgG (Cat. No sc-2025) was obtained from Santa Cruz Biotechnology, Santa Cruz, CA, USA.

### Isolation of human neutrophils

Neutrophils were isolated from heparinized human blood of healthy donors. In a first step, red blood cells were removed with a solution containing 12% (w/v) OptiPrep and 16.6 g/l methocel in 130 mM NaCl [26]. The leukocyte-rich plasma obtained from 30 ml blood was centrifuged and resuspended in 4 ml PBS followed by application to a Percoll step gradient. This gradient consisted of 72% (vol/vol) Percoll (Amersham) in PBS overlayered with 61.6% Percoll in PBS. This gradient was centrifuged at 900×g for 25 min. The neutrophil-rich band was recovered at the interface of the 72% and the 61.6% Percoll layer and washed twice with Gey's solution. The isolated cell population contained >95% neutrophils.

### HL-60 cells: culture, differentiation and transfection

HL-60 promyelocytic leukemia cells (American Type Culture Collection, ATCC) were cultured as described [27]. For differentiation, 260 µl dimethylsulfoxide was added to 8×10^6^ cells in 20 ml of Iscove's medium followed by incubation for 5 days in a humidified atmosphere at 37°C without changing the medium. For transfection, 4×10^6^ differentiated HL-60 cells were resuspended in 100 µl Nucleofector V solution diluted 2∶1 with PBS (Amaxa, Köln, Germany) and 2 µg of plasmid DNA were added. Then, the cell suspension and the plasmid DNA were transferred to a cuvette and Nucleofection was carried out (Amaxa Nucleofector, program T19). Immediately, 500 µl of medium with 20% FCS was added and the cells were transferred to a prewarmed 12-well plate containing 2.5 ml of medium with 20% FCS, followed by incubation at 37°C in a CO_2_ incubator for 3 h. Transfected cells were then washed and resuspended in Gey's containing 2% BSA.

### Murine bone marrow neutrophils: isolation and stimulation

All animal work was performed with approval from the Office for Agriculture and Nature of the Canton of Bern, Veterinary Service (project No 47/08). The wild type and PSGL-1 deficient SJL mice, previously described in [25], were euthanized, the tibia and femur dissected and flushed with cold HBSS using a 30-gauge needle. Cells were dispersed using an 18-gauge needle, pelleted, and resuspended in 5 ml HBSS. After having been passed through a 70-µm cell sieve, cells were overlayed on 5 ml of histopaque 1083, and centrifuged for 30 min at 700×g without brake. Neutrophils and red blood cells were collected at the bottom of the centrifuge tube. Erythrocytes were lysed using ACK buffer (150 mM NH_4_Cl, 10 mM KHCO_3_, 100 µM EDTA) and washed with HBSS. Neutrophils were then resuspended in 37°C RPMI media supplemented with 25 ng/ml recombinant murine G-CSF and kept for 24 h at 37°C, 5% CO_2_. After 24 h, neutrophils were washed, resuspended in HBSS, pre-incubated for 15 min at 700 rpm, 37°C, stimulated with 1 µM fNLPNTL and fixed with 10% TCA (see below).

### Immunofluorescence staining

After incubation of cells in Gey's buffer at 37°C with agitation (750 rpm) without or with inhibitors and stimuli (4×10^6^ cells/ml, 500 µl per assay) as indicated, cells were fixed with 10% trichloroacetic acid (TCA) prewarmed at 37°C for 5 min. Cells then were washed with phosphate-buffered saline (PBS), permeabilized with lysolecithin (60 µg/ml) for 10 min at RT and washed with PBS. Cells were cytocentrifuged and blocked for 1 h with blocking buffer (PBS containing 5% BSA and 10% normal goat serum). Subsequently cells were incubated with the primary antibody (rat anti-murine PSGL-1 undiluted, rabbit anti-flotillin-2 or anti-flotillin-1: both diluted 1∶100; murine anti-flotillin-2, anti-CD43 or anti P-ERM, anti-actin II, murine anti- human PSGL-1: all diluted 1∶400) in PBS, 0.5% BSA at room temperature for 1 h, rinsed once with TBST (50 mM Tris, pH 7.4, 150 mM NaCl, 0.05% Tween) and twice with PBS, blocked again for 15 min with blocking buffer. Cells were then incubated with the appropriate secondary antibodies (Alexa 488-conjugated goat-anti-rabbit or goat anti-mouse IgG antibodies diluted 1∶1500) at room temperature for 1 h, followed by washing once with TBST and twice with PBS.

Images were taken with a epifluorescence microscope (Nikon Eclipse TE 2000-U) connected to a Nikon digital camera (DXM1200F).

### Immunoprecipitation

Immediately after isolation, neutrophils used for the immunoprecipitation were incubated with diisopropylfluorophosphate (5 mM, final) in Gey's buffer (Sigma, St. Louis, MO, USA) for 5 min on ice and then resuspended in Gey's solution before being subjected to incubation. After incubation without or with fNLPNTL as indicated, 10–15×10^6^ cells per sample were centrifugated at 37°C, resuspended in 1 ml lysis buffer prewarmed at 37°C (0.5% NP-40, 50 mM NaCl, 50 mM Tris, 50 mM NaF, 30 mM sodium pyrophosphate, 2 mM sodium orthovanadate, protease inhibitor cocktail, pH 8), sonicated (88 J) and incubated on ice for 15 min. Cell lysates were then clarified by centrifugation at 12'000×g at 4°C for 5 min and pre-incubated under gentle rocking for 2 h with unconjugated protein A or protein G beads. Beads were removed and lysates incubated overnight with antibodies (2 µg, rabbit anti-flotillin-2 or murine anti-PSGL-1). Unconjugated protein A or protein G beads were then added to the lysates and incubated at 4°C for 2 h. Beads were washed 3 times with lysis buffer and boiled for 3 min in sample buffer (1% SDS, 50 mM dithiothreitol, 15% glycerol, 62.5 mM Tris/HCl (pH 6.8), 0.001% bromphenol blue). Samples were loaded without the beads on a 7.5% SDS-polyacrylamide gel, electrophoresed and blotted to a nitrocellulose membrane using a Genie blotter (Idea Scientific, Minneapolis, MN). The blots were incubated for 1 h in a blocking buffer (TBST, 5% defatted milk powder) followed by overnight incubation at 4°C with the indicated antibodies diluted in PBS containing 5% BSA and 0.02% NaN_3_. After 5 washes with TBST, the blots were incubated for 1 h with corresponding second goat-antibodies against rabbit or mouse IgG coupled to horseradish peroxidase. Detection was performed with an ECL Western detection system.

### Flotillin-2-EGFP and flotillin-1-mCherry plasmids

Total mRNA was extracted from HEK293 cells using the Promega kit. cDNA was produced by using Oligo-dT (Invitrogen) and Superscript II reverse transcriptase (Invitrogen). Full-length flotillin-2 (NCBI accession No. NM_004475) and full-length flotillin-1 (NBCI accession No. NM_005803) in HEK 293 cDNA were amplified by PCR using the Phusion DNA Polymerase (Finnzymes, Espoo, Finland, catalog No F-530) and specific primers containing XhoI (forward primers) or EcoRI (reverse primers) restriction sites. For the reverse primers, the stop codon was removed and a single base was added (in order to be in frame with the EGFP or the Cherry tag after cloning them in pEGFP-N1 or pmCherry-N1 vector). PCR products were purified on Low Melting Agarose Gel (AppliChem A3762) and cloned into a TA vector (pGEM-Easy vector Promega A1360 or pDrive vector Qiagen 231122). A-addition was performed (Qiagen A-Addition Kit 231994). Clones were transformed in DH5α competent cells, amplified and sequenced. Inserts were cut out of the TA vector using XhoI/EcoRI restriction sites and cloned into the pEGFP-N1 or pmCherry-N1 vector (ClonTech Laboratories) using the same restriction sites and sequenced.

### Statistics

Differences between data were analysed with the Student's t test, with a *P* value<0.05 considered significant. Data correspond to the mean±s.e.m.

## Results

### Flotillin-1 and -2 interact and redistribute rapidly to the uropod upon stimulation of neutrophils

**Figure 1:**
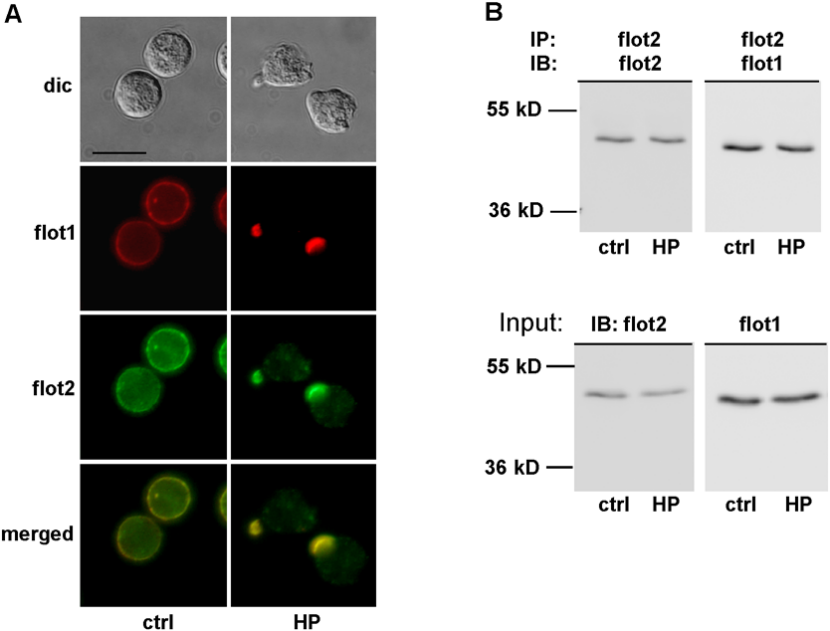
Flotillin-1 and -2 interact and redistribute to the uropod in stimulated human neutrophils. (A) Cells were incubated either in Gey's medium for 25 min at 37°C (ctrl) or were stimulated with 1 nM fNLPNTL (HP) for 5 min after a 20 min preincubation. Cells were then fixed with 10% TCA and double-labeled for flotillin-1 (flot1; mouse Ab) and flotillin-2 (flot2; rabbit Ab). Dic; differential interference contrast. Bar, 10 µm. (B) Cells were treated as in (A), lysed and subjected to immunoprecipitation (IP), using a flotillin-2 rabbit antibody. Immunoblots (IB) of the immunoprecipitated material were probed for flotillin-1 (mouse Ab) or, after stripping of the blot, for flotillin-2 (mouse Ab). Comparable amounts of both proteins were present in immunoprecipitates of resting and stimulated cells (upper panels). Three percent input lysates were also analyzed for the presence of flotillin-1 and -2 showing that equal amounts of these proteins were present in the lysates of resting and stimulated cells before immunoprecipitation (lower panels).

Human neutrophils stimulated in suspension with the chemotactic bacterial peptide fNLPNTL formed within seconds F-actin-rich ruffles. After five min of stimulation, neutrophils were fully polarized, featuring a protruding actin-rich leading edge and a contracted uropod (Fig. 1A). Flotillin-1 and -2 are ubiquitously isolated in membrane rafts/DRMs and have been postulated to organize membrane microdomains [17], [28]. We were interested to follow reorganization of membrane microdomains during neutrophil stimulation. We therefore assessed the re-distribution of the raft markers flotillin-1 and -2 during neutrophil activation.

**Figure 2:**
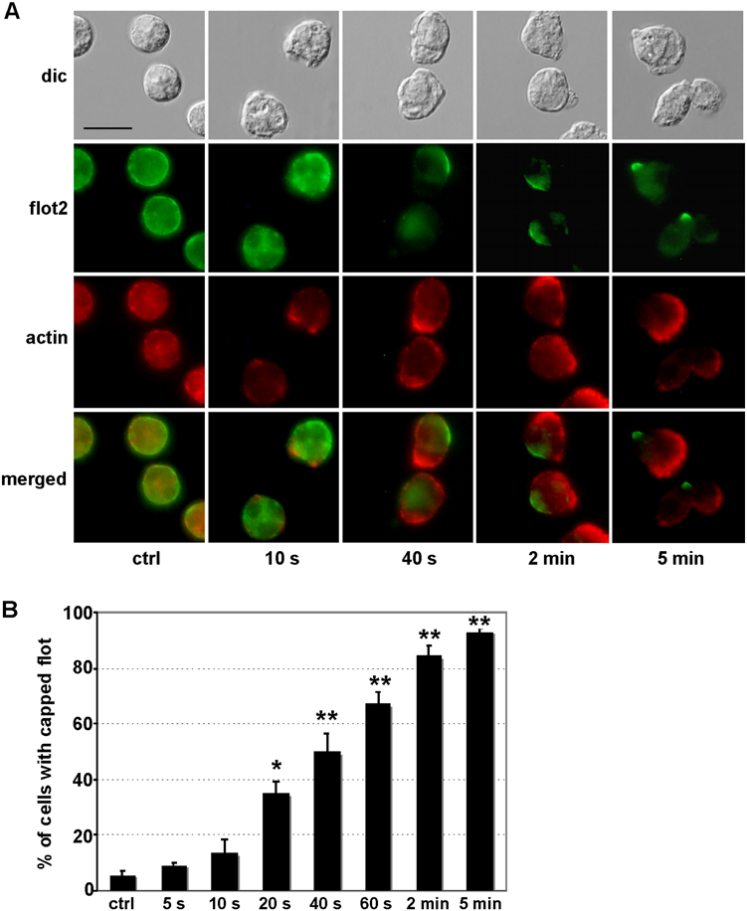
Fast capping and redistribution of flotillin-2 to the uropod in stimulated human neutrophils. (A) Cells were either incubated in Gey's medium for 25 min at 37°C (ctrl) or were stimulated with 1 nM fNLPNTL for the indicated times. Incubation was stopped by fixation with 10% TCA. Fixed cells were double-labeled for flotillin-2 (flot2; rabbit Ab) and actin (mouse Ab). Bar, 10 µm. (B) The percentage of cells treated as described in panel (A) displaying a flotillin-2 cap was determined by visual counting. 100 cells were inspected per experiment (mean±s.e.m. of 4 independent experiments). Significant differences compared to unstimulated controls are marked: * P<0.001, ** P<0.0001.

Endogenous flotillin-1 and -2 colocalized both in resting and stimulated cells (Fig. 1A) and flotillin-1 was co-immunoprecipitated by a flotillin-2 antibody (Fig. 1B), indicating that flotillin-1 and -2 interact and likely form hetero-oligomers in human neutrophils. Interaction of flotillin-1 and -2 was not modified by stimulation of neutrophils with the chemotactic peptide fNLPNTL (Fig. 1B). Flotillins were linearly associated with the plasma membrane of resting cells with some enrichment in small aggregates and formed very distinct caps at the tip of uropods of polarized cells (Fig. 1A). In order to determine if the capping preceded or followed uropod formation, we analyzed the time course of flotillin-2 capping in cells stimulated with fNLPNTL. The linear membrane association of flotillin-2 in resting cells was impaired already 10 s after stimulation and the number of cells bearing a flotillin cap was significantly increased when compared to controls 20 s (*P*<0.001) after stimulation. Half-maximal effects on capping were observed at 40 s (Fig. 2A,B). Formation of F-actin-rich ruffles at one side of the cell correlated with redistribution of flotillin-2 (Fig. 2A). At any time point, the flotillin caps were located opposite to the F-actin-rich ruffles. Thus, the capping of flotillin-2 preceded uropod formation, possibly marking the site of the future uropod in stimulated neutrophils. Comparable results were obtained for flotillin-1 (data not shown).

### Overexpression of tagged flotillin-1 or -2 alone in neutrophil-like HL60 cells does not result in capping of the exogenously expressed proteins whereas simultaneous overexpression results in enrichment of tagged flotillins in the uropod

**Figure 3:**
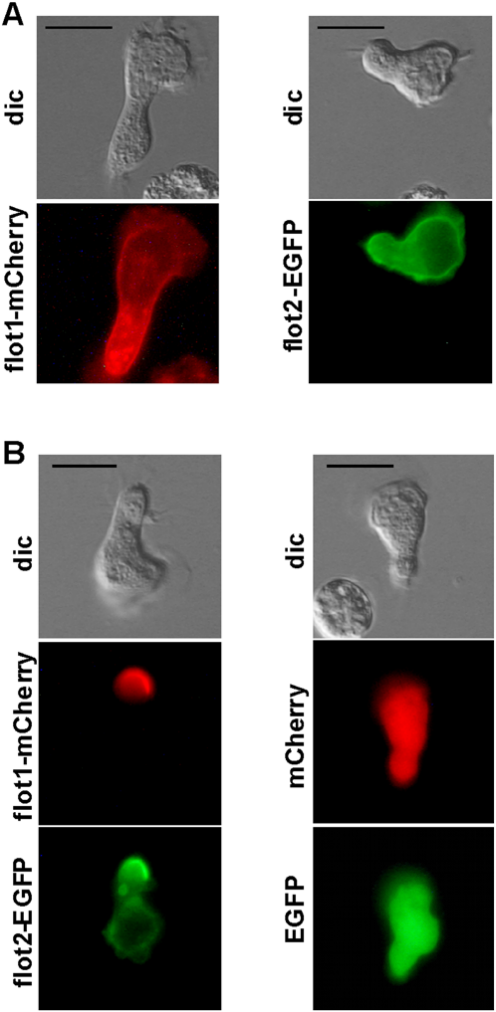
Co-expression of flotillin-1-mCherry and flotillin-2-EGFP in dHL-60 cells promotes uropod enrichment of tagged flotillins. dHL-60 cells were transfected with vectors encoding either flotillin-1-mCherry (flot1-mCherry) or flotillin-2-EGFP (flot2-EGFP) alone (A) or were cotransfected with both plasmids or with pmCherry and pEGFP (B), as indicated and stimulated with 10 nM fNLPNTL for 10 min. Living cells were then placed on the stage of a microscope heated at 37°C and photographed. Bar, 10 µm.

Primary human neutrophils are notoriously difficult to transfect. Therefore, DMSO differentiated HL60 cells (dHL-60 cells), which display comparable features and migratory behavior as primary neutrophils and which are transfectable, are used as a model system for neutrophil studies [27], [29]. However, whereas 93%±1% (n = 3) of stimulated neutrophils bear endogenous flotillin-1 and -2 caps, such an endogenous flotillin staining pattern could be observed in only 13%±1% (n = 3) of stimulated dHL60 cells. PSGL-1 co-capped with flotillins in approximately 70% of the cells with flotillin caps. Comparable to neutrophils, caps in dHL-60 cells contain both flotillin-1 and -2 (data not shown). Ectopically expressed flotillin-1 tagged with mCherry at the C-terminus showed a linear membrane association in dHL-60 cells and was also present in cytosolic granules (Fig. 3A). Cap formation of exogenously expressed flotillin-1 was very rarely observed (not shown). Flotillin-2 tagged with EGFP at the C-terminus showed a similar location (Fig. 3A), whereas EGFP or mCherry expressed alone or co-transfected showed a diffuse cytosolic location (Fig. 3B). Interestingly, co-transfection of both tagged flotillin isoforms in dHL60 cells dramatically enhanced formation of caps containing the tagged proteins in the uropod of 98%±2% (n = 3) of the co-transfected polarized cells (Fig. 3B). Those results show that flotillins ectopically expressed in dHL-60 cells locate in the same cellular area as endogenous flotillins in human neutrophils and suggest that flotillin-1 and -2 collaborate to form membrane microdomains in the uropod of polarized neutrophils.

### Flotillin capping requires the integrity of the actin cytoskeleton but does not rely on actin-myosin contractions in chemotactic peptide-stimulated cells

**Figure 4:**
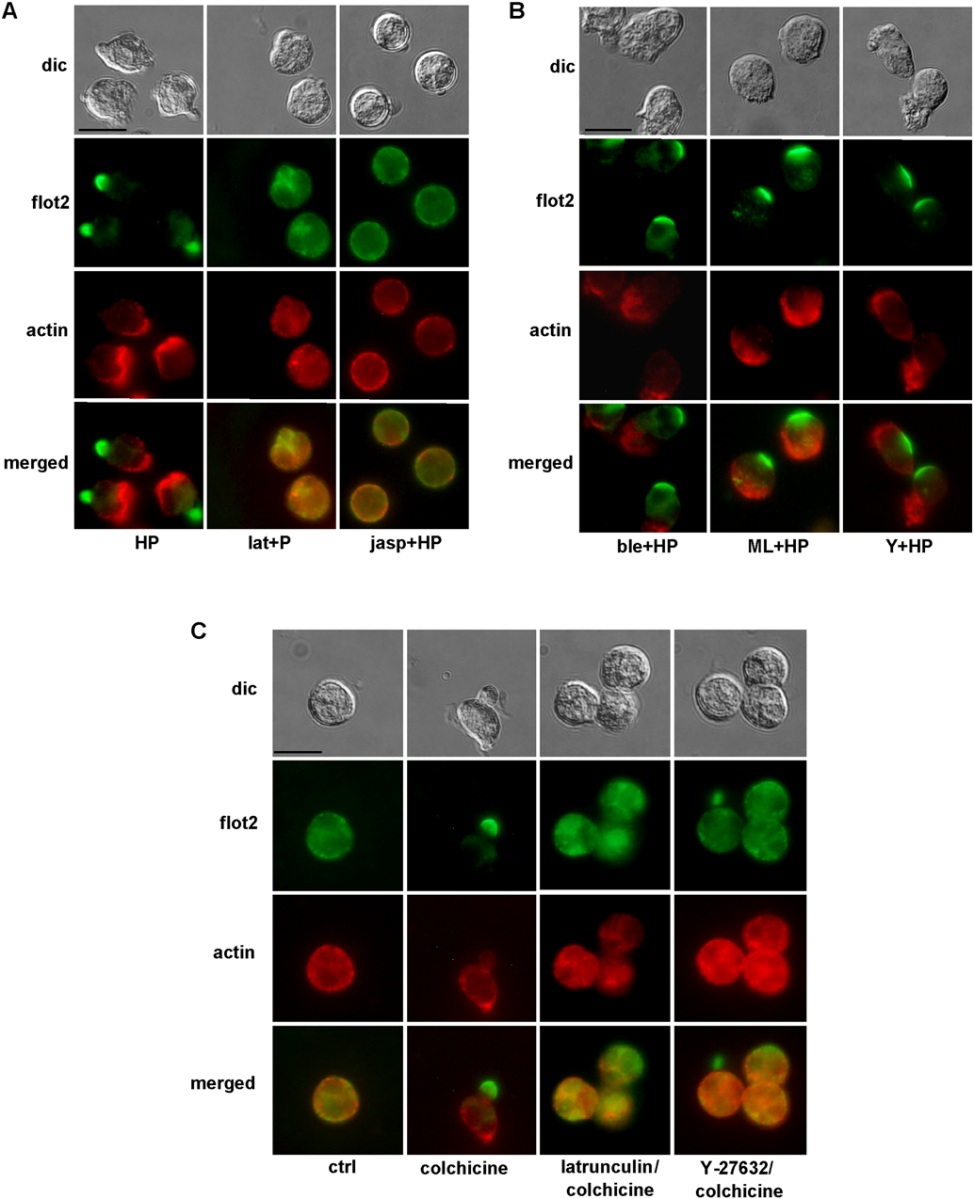
Impact of agents that affect F-actin, myosin II or microtubules on flotillin-2 capping. Human neutrophils were either incubated in Gey's medium for 30 min at 37°C (ctrl) or were preincubated for 20 min without or with (A,C) latrunculin A (lat; 1 µM) or (A) jasplakinolide (jasp; 5 µM) or (B) blebbistatin (ble; 100 µM), or ML-7 (ML; 10 µM), or (B,C) Y-27632 (Y; 10 µM) respectively followed by stimulation with 1 nM fNLPNTL (HP) for 10 min (A,B), or were incubated for 30 min with 10 µM colchicine as indicated (C). Cells were then fixed with 10% TCA and double-labeled for flotillin-2 (rabbit Ab) and actin (mouse Ab). Bar, 10 µm.

The actin cytoskeleton is required for organization and function of membrane microdomains in lymphocytes [14], [15] and for redistribution of DRM proteins in the uropod of leukocytes [8], [10]. As it has been shown recently that flotillin-2 can directly bind actin [23], we explored the contribution of the actin cytoskeleton to the capping of flotillin isoforms. Disruption of the actin cytoskeleton with latrunculin A treatment as well as its stabilization by jasplakinolide completely suppressed formation of the endogenous fNLPNTL-induced flotillin-2 caps in primary neutrophils (Fig. 4A), showing that actin cytoskeleton integrity and dynamics were required for flotillin capping. Flotillin-2 showed a diffuse cytosolic location in cells pretreated with latrunculin A, but was still membrane-associated after treatment with jasplakinolide. Actin also showed some membrane-association in jasplakinolide-treated cells but did not markedly colocalize with flotillin-2 (Fig. 4A). Comparable data were obtained for flotillin-1 (data not shown).

Actin-myosin contractions are responsible for the formation of the uropod in neutrophils [12], [30]. Inhibition of Rho-kinase by Y-27632 or of myosin light chain kinase (MLCK) by ML-7 or inhibition of myosin activity by blebbistatin abolished formation of a contracted tail but still allowed formation of actin-rich ruffles in primary human neutrophils (Fig. 4B). These inhibitors also had no impact on the capping of endogenous flotillin-2 (Fig. 4B) and flotillin-1 (not shown) opposite of the actin-rich ruffles.

Rear signaling based on Rho/Rho-kinase activation and myosin II contractility leading to the formation of the uropod can be selectively activated by depolymerization of the microtubule network, without activation of front signaling molecules such as phosphatidylinositol 3-kinase, and results in development of front-tail polarity and migration in neutrophils [31], [32]. Disruption of the microtubule network in neutrophils with colchicine induced flotillin-2 capping in the uropod of primary neutrophils identical to findings with fNLPNTL stimulated cells (Fig. 4C). Comparable data were obtained for flotillin-1 (not shown). Interestingly, flotillin capping induced by colchicine could be abolished both by latrunculin and by Rho-kinase inhibition (Fig. 4C). These findings indicate that Rho-kinase activation is only required for colchicine-induced but not for chemotactic peptide-induced flotillin capping; at the same time both stimuli require actin polymerization.

### Flotillin-2 redistribution in the uropod precedes that of CD43 and P-ERM

**Figure 5:**
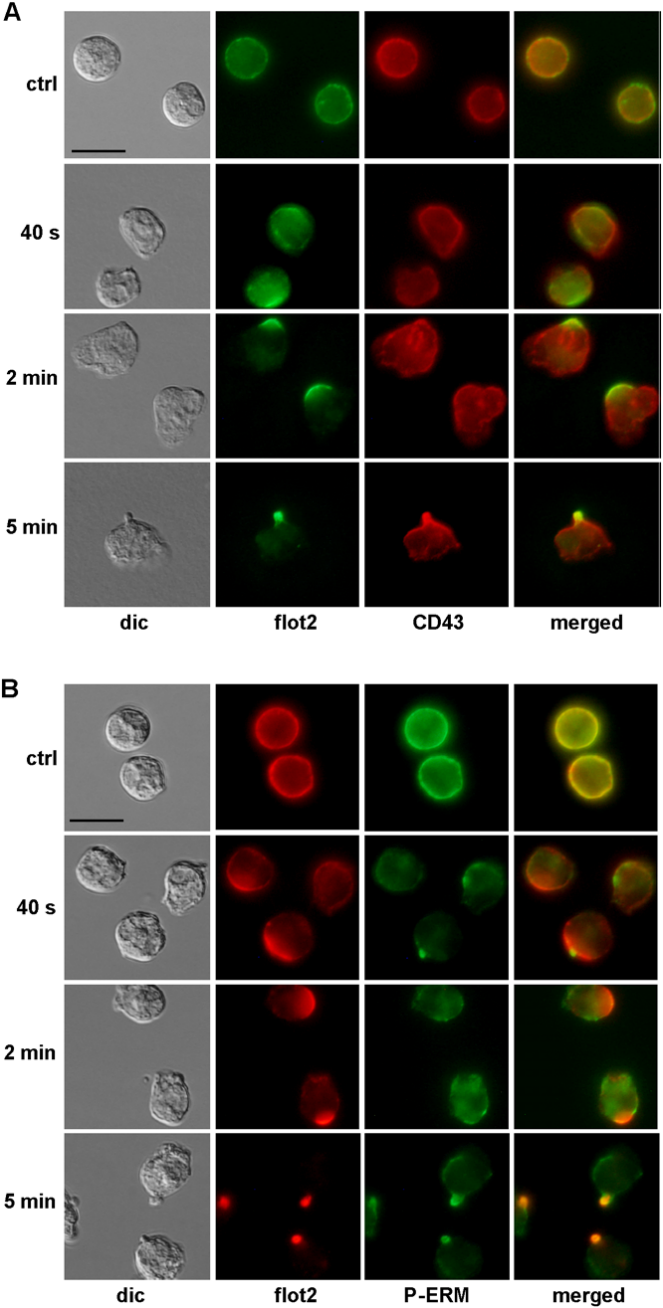
Flotillin-2 capping precedes that of CD43 or P-ERM during polarization of human neutrophils. Cells were either incubated in Gey's medium for 25 min at 37°C (ctrl) or were stimulated with 1 nM fNLPNTL for 40 s, 2 or 5 min. Incubation was stopped by fixation with 10% TCA. Fixed cells were double-labeled (A) for flotillin-2 (flot2; rabbit Ab) and CD43 (mouse Ab) or (B) for flotillin-2 (flot2; murine Ab) and P-ERM (rabbit Ab). Bar, 10 µm.

Activated ERM proteins and the adhesion protein CD43 are known to redistribute and cap in the uropod of polarized neutrophils [12], [33]. The highly homologous active ERM proteins are phosphorylated on a C-terminal threonine and can be recognized using a specific anti-P-ERM antibody which does not discriminate between the three isoforms. To determine if flotillin capping and redistribution were related to those of P-ERM and CD43, we performed immunostaining of primary neutrophils incubated with fNLPNTL for 5 s to 5 min. We compared the staining for flotillin-2 with that for CD43 and P-ERM respectively. As shown in Fig. 2 and Fig. 5, flotillin cap formation preceded uropod formation and could be clearly observed 40 s after addition of chemotactic peptide. The percentage of cells bearing a flotillin cap had reached 84%±4% after 2 min. In contrast, P-ERM and CD43 caps were almost not detectable at earlier time points than 5 min and capping of those proteins occurred only in cells with a fully formed uropod (Fig. 5A,B). Thus, although localization of flotillin, P-ERM and CD43 are similar in resting and fully polarized cells, the stimulus-dependent redistribution of flotillin was much faster when compared to that of P-ERM and CD43. These results suggest that the molecular mechanisms responsible for organizing flotillins on one hand, and P-ERM and CD43 on the other hand, are different. Note that P-ERM staining was decreased after addition of chemoattractant (Fig. 5B), correlating with data obtained with immunoblotting [33].

### PSGL-1 co-redistributes with flotillins and flotillins co-immunoprecipitate with PSGL-1

**Figure 6:**
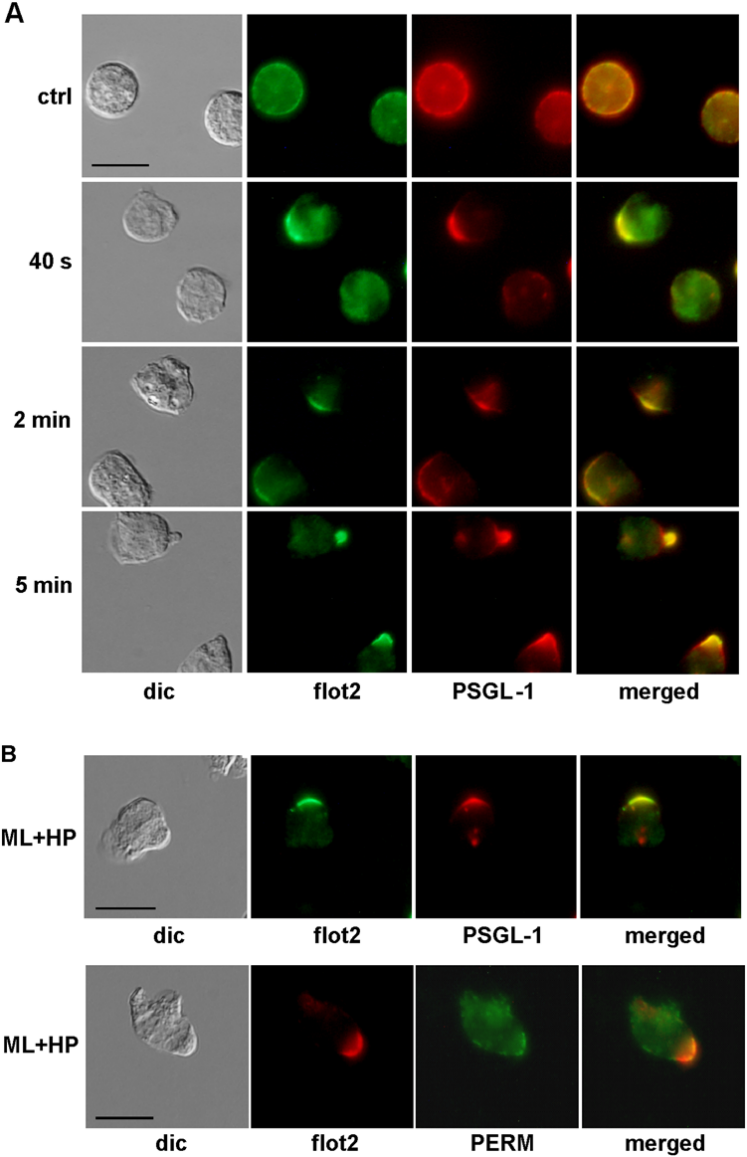
Coordinated redistribution and capping of flotillin-2 and PSGL-1 during polarization of human neutrophils. (A) Cells were either incubated in Gey's medium for 30 min at 37°C (ctrl) or were stimulated with 1 nM fNLPNTL (HP) for 40 s, 2 or 5 min. (B) Cells were preincubated for 20 min with 10 µM ML-7 (ML) followed by stimulation with 1 nM fNLPNTL (HP) for 10 min. (A,B) Incubation was stopped by fixation with 10% TCA and fixed cells were double-labeled (A,B) for flotillin-2 (flot2; rabbit Ab) and PSGL-1 (murine Ab) or (B) for flotillin-2 (murine Ab) and P-ERM (PERM; rabbit Ab). Bar, 10 µm.

The selectin ligand PSGL-1 is another adhesion protein that forms a cap in the uropod of polarized neutrophils [13]. We compared flotillin-2 relocalization with that of PSGL-1 upon primary neutrophil stimulation using immunofluorescence staining of endogenous proteins. In resting cells, both proteins colocalized at the plasma membrane. Comparable to flotillin, PSGL-1 started to cap 20 to 40 s after addition of fNLPNTL, and 84%±4% (n = 3) of the cells displayed PSGL-1 capping after 2 min (Fig. 6A). Caps of PSGL-1 co-localized with caps of flotillin-2 in the uropods of 99%±0% (n = 3) of the observed polarized cells (Fig. 6A). Moreover, PSGL-1 caps were insensitive to inhibition of uropod formation by the MLCK inhibitor ML-7, comparable to flotillin caps (Fig. 6B). In contrast, P-ERM capping (Fig. 6B) and CD43 capping (data not shown) were disturbed by this treatment, indicating again different mechanisms of capping of flotillin as compared to formation of CD43 and P-ERM caps.

**Figure 7:**
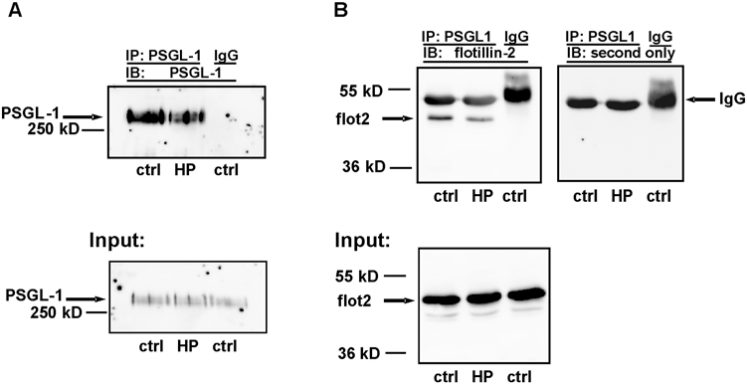
Co-immunoprecipitation of flotillin-2 by a PSGL-1 antibody. Cells were incubated either in Gey's medium for 30 min at 37°C (ctrl) or were stimulated with 1 nM fNLPNTL (HP) for 10 min after a 20 min preincubation. Cells were then lysed and subjected to immunoprecipitation (IP), using a murine PSGL-1 antibody or a murine IgG as control, as indicated. Upper parts of the immunoblots (IB) were probed for PSGL-1 (A) (murine Ab) and lower parts of the blots for flotillin-2 (B) (flot2, murine Ab; left upper and lower panels in B). The band of approximately 50 kD detectable in the immunoprecipitates just above flotillin (upper panels in B) corresponds to the heavy chain of IgG, as it also appeared when the blot was stripped and decorated only with the secondary anti-murine IgG antibody (upper right panel in B). Three percent input lysates were in addition analyzed for the presence of PSGL-1 (A) and flotillin-2 (B) showing that equal amounts of these proteins were present in the lysates of resting and stimulated cells before immunoprecipitation (lower panels in A and B). Data representative of 3 independent experiments are shown.

Next, we performed immunoprecipitation of primary neutrophil lysates using a monoclonal anti-PSGL-1 antibody in order to determine if the identical capping time course and co-localization of flotillins and PSGL-1 were the result of a direct or indirect interaction between those two proteins. As shown in Fig. 7, the murine antibody to PSGL-1 precipitates PSGL-1 (A) and co-immunoprecipitates flotillin-2 both from lysates of resting and of activated cells (B). We used murine antibodies both for immunoprecipitation of PSGL-1 and immunoblotting of flotillin-2. This resulted in detection of the IgG heavy chain band located just above the flotillin band by the second antibody (see Fig. 7B). The flotillin-2 band was not detectable when control murine IgG was used instead of the specific antibody for immunoprecipitation (Fig. 7B). The amount of flotillin-2 associated with PSGL-1 was not significantly modified by activation of neutrophils by fNLPNTL. Similar results were obtained by probing the blot with a murine anti-flotillin-1 antibody (n = 2, data not shown). Thus, flotillin-1 and -2 and PSGL-1 interact (directly or indirectly) in resting and in polarized neutrophils and show coordinated redistribution upon chemotactic stimulation.

### Deficiency in PSG-1 does not prevent capping of flotillins

**Figure 8:**
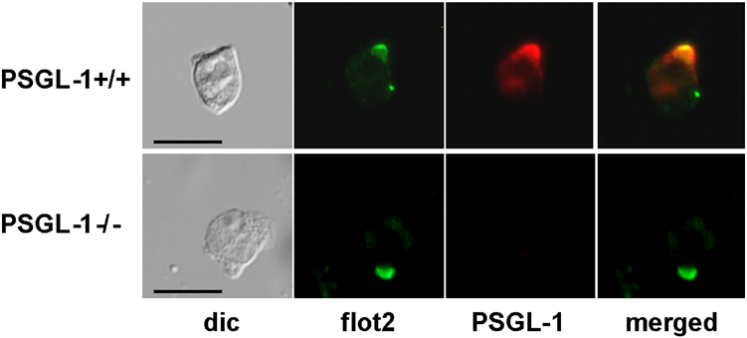
Capping of flotillin-2 in PSGL-1 deficient bone marrow neutrophils. After isolation and 24 h maturation with 25 ng/ml G-CSF, murine bone marrow neutrophils derived from wild type or PSGL-1−/− mice were resuspended in HBSS media, pre-incubated for 15 min at 37°C and incubated for 3–5 min with 1 µM fNLPNTL. Incubation was stopped by fixation with 10% TCA and fixed cells were double-labeled for flotillin-2 (flot2; rabbit Ab) and PSGL-1 (rat Ab). Bar, 10 µm.

The close correlation of flotillins and PSGL-1 reorganization prompted us to investigate whether PSGL-1 is required for coalescence of flotillin-rich microdomains using neutrophils derived from PSGL-1−/−mice. In murine wild type neutrophils flotillin-1 and-2 colocalized in the uropod (data not shown). Moreover PSGL-1 colocalized with flotillins in the uropod caps (Fig. 8), comparable to our observations with human neutrophils (Fig. 6). However, capping of flotillin-2 was undisturbed by the absence of PSGL-1 (Fig. 8), demonstrating that PSGL-1 does not play a significant role in relocating flotillin containing microdomains at the uropod of polarized neutrophils. Comparable results were obtained for flotillin-1 (data not shown).

## Discussion

Flotillin-1 and -2 are two highly homologous proteins, which associate with the inner leaflet of the plasma membrane via hydrophobic regions and myristoylated and palmitoylated residues [17], [34]. They belong to the SPFH (stomatin/prohibitin/flotillin/HfLK/C) domain-containing protein family, whose members are characterized by a ubiquitous enrichment in detergent resistant membranes (DRMs) and a propensity to oligomerize [18]. Depending on the cell type, flotillin-1 and -2 can have different or identical subcellular localization. Flotillins assemble in membrane microdomains [35], [36] and form homo and heterotetramers [28]. Coexpression of flotillin-1 and -2 is sufficient to create membrane microdomains in COS-7 and HeLa cells [37]. Those properties make flotillins good candidates for scaffolding and defining functional membrane microdomains. In resting lymphocytes, flotillin-1 and -2 form stable, asymmetrically located caps, and activation of lymphocytes results in the recruitment of signaling proteins into those caps [38], [39]. Our data now suggest an important structural role of flotillins in the formation and/or stabilization of uropods of stimulated neutrophils. Moreover we report novel interactions of flotillins with the selectin-ligand PSGL-1.

### Flotillins cap very early in chemotactic peptide-stimulated neutrophils dependent on actin turnover but independent of myosin contractility

Our results show that endogenous flotillin-1 and -2 colocalize and interact in resting and stimulated neutrophils (Fig. 1), likely forming heteroligomers. Flotillin-1 and -2 in resting neutrophils are evenly distributed along the cell periphery and are organized in small aggregates, in agreement with their raft location (Fig 1A). Larger flotillin caps appear only in stimulated cells (Fig 1A and 2). Formation of these caps is a very early event during cell stimulation, occurring 20 to 40 s after addition of stimulus and occurs in more than 90% of the primary neutrophils (Fig. 2). Flotillin caps always form opposite of actin-rich ruffles where later on the uropod is formed. Our results strongly suggest that these caps mark the site of the future uropod. Organization of flotillin in such a locally confined structure suggests a stimulus-induced coalescence of flotillin-containing membrane microdomains into a larger platform. Both flotillin-1 and flotillin-2 are involved in the formation of this macrodomain, as uropod location of exogenously expressed flotillins is dramatically enhanced by co-overexpression of tagged flotillin-1 and -2 in dHL-60 cells as compared to cells expressing only one tagged isoform (Fig. 3A,B). These data show that overexpression of both flotillin 1 and 2 is sufficient to induce formation of specific membrane domains in dHL-60 cells. Furthermore our results demonstrate that ectopically expressed flotillins are located in the same place in dHL-60 cells, the uropod, comparable to the location of endogenous flotillins in human neutrophils. dHL-60 cells are thus a good model system for the study of the dynamic behaviour of expressed flotillins during cell migration, even if uropod capping of endogenous flotillins is much less pronounced as compared to human neutrophils. Our findings are in accordance with the results obtained with HeLa cells, where co-overexpression of both flotillins is required for the formation of membrane domains [37].

Membrane microdomains and clustering-dependent installation of signaling platforms in the plasma membrane are thought to be involved in establishment of leukocyte polarity. Disruption of cholesterol-rich membrane microdomains with methyl-β-cyclodextrin suppresses induction of polarization and migration of neutrophils and differentiated HL-60 cells [6], [7]. Stimulus-induced polarization results in segregation of proteins retrieved in DRMs to leading and trailing edges [8], [10]. Proteins such as ICAMs, selectins, PSGL-1, CD43 or CD44, which reside in membrane microdomains, are enriched in the uropod, similar to our findings for flotillin. Capping of these proteins is abolished by depolymerization of the actin cytoskeleton [12], [13]. Similarly cholesterol-rich microdomains in lymphocytes are linked to the actin cytoskeleton and depend on it for their integrity [14], [15].

We show now in Fig. 4A that stimulus-dependent capping of flotillins in human neutrophils requires integrity and dynamics of the actin cytoskeleton. Our findings on the actin-dependence of flotillin-1 and -2 reorganization in neutrophils correlates with recent findings, showing the direct association of flotillin-2 with actin [23]. Double-staining for flotillin-2 and actin in stimulated cells shows that, in agreement with actin-dependence of flotillin-raft coalescence, the time course of flotillin reorganization parallels that of actin (Fig. 2A). However, actin accumulates in ruffles opposite to flotillin caps within 1 to 2 min of stimulation (Fig. 2A). The merge of actin and flotillin stainings shows clearly that flotillin is not present in actin-rich regions of the cells (Fig. 2A). Actin polymers can be organized in different ways, forming different pools of F-actin, and different actin antibodies can visualize different actin patterns [40]. Phalloidin staining of polarized neutrophils indicates that small amounts of F-actin are also present in the contracted tail of neutrophils [30]. However TCA fixation of cells that is required for optimal flotillin immunofluorescence does not allow staining of F-actin with phalloidin. Interestingly, according to recent work by Cooper et al. [41], the neutrophil uropod contains a subpopulation of F-actin which can be visualized with an F-actin probe that detects a more stable actin population. A possible mechanism of formation of the flotillin platforms in stimulated cells could involve stimulus-dependent rapid establishment of the stable F-actin pool, which segregates into areas that later develop into the uropod. Direct connections of flotillins with this F-actin pool would then power their relocation and capping in the uropod. However such a mechanism would require specific interactions of flotillin with stable F-actin but not with the dynamic F-actin present in the front of the cell. Evidence for such specific interactions is yet lacking.

We show moreover that Rho-kinase dependent signaling, leading to acto-myosin contraction and uropod formation in stimulated neutrophils, is in contrast not required for chemotactic peptide-induced flotillin capping (Fig. 4B), in accordance with the fact that flotillin capping occurs several minutes before the appearance of the uropod (Fig. 2). We cannot exclude that Rho alone, via effectors different from Rho-kinase, might influence flotillin organization. However this is unlikely, as there is to our knowledge no evidence in the literature of Rho signaling acting separately of Rho-kinase in the context of neutrophils polarization and migration. Induction of neutrophil polarization via microtubule disassembly [31] also induces flotillin capping which now can be suppressed by Rho-kinase inhibition (Fig. 4C). Thus activation of rear signaling, correlating with formation of the uropod, appears to be sufficient in colchicine-treated cells but is not necessary for flotillin capping in fNLPNTL-stimulated cells. Note that actin assembly is required for flotillin capping both in cells stimulated by fNLPNTL and by microtubule disassembly (Fig. 4). Our findings suggests that several parallel redundant pathways may promote formation of flotillin scaffolds in neutrophils.

### Selective interaction of flotillin-1 and -2 with PSGL-1

**Figure 9:**
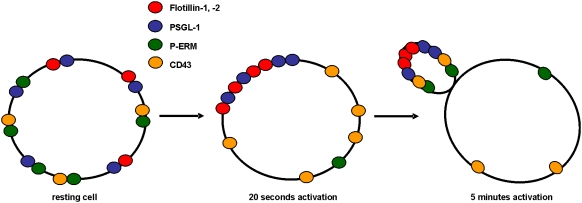
Schematic representation of reorganization of flotillins, PSGL-1, P-ERM and CD43 during neutrophil activation. Resting cells exhibit randomly dispersed small rafts at the plasma membrane containing flotillins and/or PSGL-1 and/or P-ERM and/or CD43. 20 s after addition of chemotactic stimulus, flotillins and PSGL-1 co-assemble into large caps at the site of the future uropod, whereas P-ERM and CD43 still are randomly distributed at the plasma membrane. ERM phosphorylation is decreased. These flotillin/PSGL-1 caps persist and may mark the site of the future uropod. 5 min after the onset of activation, flotillins, PSGL-1, CD43 and P-ERM all co-cap in the uropod of polarized cells.

In lymphocytes, ERM proteins are located in DRMs and associate with actin on one hand and with proteins belonging to membrane microdomains of the uropod on the other hand [9], [16]. Therefore, ERM proteins are postulated to link membrane microdomains to the actin cytoskeleton, supporting their formation and redistribution to the uropod upon stimulation [16]. In neutrophils, P-ERM, CD43, CD44, L-selectin and the selectin-ligand PSLG-1 have all been isolated in DRMs and shown to redistribute to the uropod of polarized cells [8], [11], [42]. However, our results show that the dynamics of redistribution of CD43 and active ERM lag behind that of flotillins (Figs. 5 and 9). Coalescence and capping of flotillins occurs very early in cells not yet fully polarized, whereas formation of caps containing P-ERM and CD43 correlates with that of the uropod (Figs. 5 and 9). Moreover, prevention of uropod formation by MLCK inhibition disturbs capping of P-ERM and CD43, but not that of flotillins (Fig. 6B).

Membrane microdomains containing CD43 and linked to the actin cytoskeleton via ERM proteins are therefore possibly not identical with flotillin-containing microdomains. Another explanation would be a transient dissociation of ERM proteins and of CD43 from flotillin-containing membrane domains during the initial phase of neutrophil activation. Indeed, transient dephosphorylation and thus inactivation of ERM has been noted in neutrophils [33]. Moreover Gupta et al. [43] have provided evidence that transient ezrin inactivation during B-cell activation allows coalescence of small rafts that are tethered to F-actin via ezrin and thus are kept apart in resting cells. Possibly, small flotillin-rich microdomains also are coupled to F-actin via ERM in resting neutrophils. Transient ERM inactivation during chemotactic stimulation may be required for release of these platforms from the cortical actin and formation of large flotillin-rich macrodomains. These macrodomains could then associate directly with the newly formed stable F-actin pool located opposite of the dynamic F-actin in the front. This hypothesis will be tested in future experiments.

Whereas the time course and the sensitivity to inhibition of myosin activity of CD43 and P-ERM capping do not correlate with those of flotillin redistribution, another raft-resident protein, PSGL-1, colocalizes with flotillins at all times assessed before and after stimulation, forms caps even in the absence of a uropod and co-precipitates with flotillins. Association of PSGL-1 with flotillins is not significantly affected by the activation state of neutrophils (Figs. 6, 7 and 9). These results strongly suggest that flotillins and PSGL-1 belong to the same membrane microdomains, which undergo coalescence into larger platforms upon stimulation. We show in Fig. 8 that the integrity and redistribution of those microdomains do not rely on PSGL-1, as capping and uropod location of flotillins is not affected in PSGL-1 deficient neutrophils.

PSGL-1-mediated adhesion to selectin on endothelial cells mediates rolling and capture of neutrophils [1], [2]. Neutrophil activation and concomitant clustering of PSGL-1 correlates with the decrease of rolling and the increase in firm adhesion and intravascular crawling [2], [13]. Our data now suggest that flotillin association with PSGL-1 might mediate actin powered relocalization of the latter molecule into the uropod of polarized neutrophils.

In lymphoid cell lines and HL-60 cells, PSGL-1 has been shown to interact with ERM proteins, and ERMs are responsible for targeting PSGL-1 to the uropod in lymphocytes [42], [44]. Based on our findings the mechanisms of targeting of PSGL-1 to the uropod may thus differ in lymphocytes and peripheral blood neutrophils. Interestingly, in agreement with our findings, a recent study on primary murine neutrophils demonstrates that the cytosolic domain of PSGL-1, which mediates ERM interaction, is not required for proper redistribution of PSGL-1 into the uropod of stimulated murine neutrophils [45]. The authors suggest that a partnership with a raft protein might be responsible for PSGL-1 redirection to the uropod [45]. In the light of our results, flotillins could be the raft molecules that relocalize PSGL-1 into the uropod of polarized neutrophils. Our findings do not exclude a later reassociation of ERM proteins with PSGL-1 in the uropod of neutrophils.

In summary our results demonstrate that flotillin-1 and -2 associate in resting and activated neutrophils. Flotillin clusters reorganize early during chemotactic peptide induced cell activation, later on forming caps at the tip of the uropods of polarized neutrophils. Formation of those caps relies on integrity and dynamics of the actin cytoskeleton but does not require Rho-kinase-dependent signaling and myosin II activation leading to uropod formation in chemotactic peptide-stimulated cells. We show that flotillins interact directly or indirectly with PSGL-1, and that redistribution of flotillin and PSGL-1 upon stimulation precedes that of the actin membrane cytoskeleton linker ERM proteins and the uropod protein CD43. Our results suggest that flotillin scaffolds are important structural features of the neutrophil uropod, localizing adhesion molecules such as PSGL-1. The precise protein arrangement in these scaffolds will be addressed in future studies.
